# Surgical Management of Ischemic Priapism: what are the New Options?

**DOI:** 10.1590/S1677-5538.IBJU.2024.0497

**Published:** 2025-01-10

**Authors:** Rohit Badia, Sidney Roberts, Alexandria Hertz, Allen Morey, Maia VanDyke

**Affiliations:** 1 University of Texas Southwestern Medical Center Department of Urology Dallas TX USA Department of Urology, University of Texas Southwestern Medical Center, Dallas, TX, USA;; 2 Urology Clinics of North Texas Dallas TX USA Urology Clinics of North Texas, Dallas, TX, USA

**Keywords:** Priapism, Erectile Dysfunction, Surgical Procedures, Operative

## Abstract

Ischemic priapism is a true urologic emergency. Prompt intervention is required to alleviate the compartment syndrome and restore perfusion to the corporal bodies; failure to do so results in irreversible damage, fibrosis, and profound erectile dysfunction. This paper's objective is to review current literature surrounding the management options for ischemic priapism, focusing on newer surgical techniques. A PubMed database search was performed in June 2024, encompassing the terms "priapism," and "surgical management." Articles were reviewed by two authors independently and included if they were deemed to pertain specifically to management of ischemic priapism. In the acute setting (certainly for priapism lasting <24 hours), management is often successful using bedside maneuvers such as aspiration, irrigation, and injection of sympathomimetic agents. For more prolonged priapism, more aggressive intervention is often warranted. Newer tunneling techniques—including penoscrotal decompression and the corporal snake maneuver—have shown promising preliminary results, not just in terms of priapism resolution but also perhaps sexual function recovery.

## INTRODUCTION

Ischemic priapism is a urologic emergency defined as a painful erection lasting >4 hours; it presents with a rigid penis with tissue hypoxia as confirmed by corporal blood gas (1). The natural sequelae of ischemic priapism are corporal ischemia, necrosis, and fibrosis, eventually culminating in erectile dysfunction (2). The longer the duration, the more challenging it becomes to treat (1). As such, prompt treatment of priapism is imperative to preserve erectile function.

At 24-36 hours after priapism onset, the rate of erectile dysfunction is around 50% (3). Medical management at this point usually fails, and surgical approaches are required (4). Currently, there is no consensus definition of "prolonged" ischemic priapism by the American Urological Society or the Sexual Medicine Society of North America (5). However, given the difference in management for long-standing priapism we herein define acute ischemic priapism as that lasting <24 hours in duration and prolonged ischemic priapism lasting ≥24 hours.

Management methods for ischemic priapism range from simple corporal aspiration and irrigation to operative intervention via shunt or penile prosthesis placement. The choice of approach depends on the priapism duration, failure of other modalities, patient's baseline erectile function, and surgeon comfort with procedures (6). In this review, we describe classic ischemic priapism management strategies and then consider more recently described surgical techniques.

## MATERIALS AND METHODS

A narrative review was carried out on the management options for ischemic priapism, with analysis of papers published over the past 50 years. A comprehensive PubMed search was performed in June 2024 using the Medical Subject Heading (MeSH) term: "priapism." Additional free-text searches were performed using the following terms: "ischemic priapism," "management," "tunneling." Articles were reviewed individually by two authors (RB and SR) for inclusion. Articles were excluded if upon review the focus was non-ischemic priapism, malignant priapism, or pediatric priapism. Non-English articles and case reports were also excluded.

## MANAGEMENT OF ACUTE ISCHEMIC PRIAPISM

Acute ischemic priapism is typically managed with a combination of corporal irrigation/aspiration and injection of sympathomimetic agents (7). After ensuring adequate local anesthesia via local block, an arterial blood gas needle is inserted into one corpus cavernosum, taking care to avoid urethral injury. Blood gas analysis showing low pO2, high pCO2, and acidic pH confirms ischemic priapism. If blood gas shows high pO2, low pCO2, and normal to slightly basic pH, this suggests nonischemic priapism, which does not routinely require intervention (8). Treatment may be offered in select cases; however, these management strategies are beyond the scope of this review.

After confirmation of ischemic priapism, a 16- or 18-gauge needle is placed into the lateral corpora. A combination of irrigation and aspiration with normal saline disrupts and evacuates the ischemic coagulum from the corporal bodies. Manual pressure on the phallus shaft may also help to decompress the clotted blood. This process typically resolves the penile compartment syndrome and allows the return of oxygenated blood, facilitating recovery (9).

Injecting a sympathomimetic agent along with aspiration and irrigation can improve chances of successful detumescence and is recommended in AUA guidelines (5). Phenylephrine, an alpha-1 agonist, is the preferred agent for priapism takedown given its lower risk for cardiotoxicity versus similar medications like epinephrine, norepinephrine, and ephedrine. Telemetry monitoring is mandated given the risk of hypertension and reflex bradycardia. These agents should be avoided in those patients with uncontrolled hypertension and monoamine oxidase inhibitor use (10). Success rate for priapism resolution is ~30% with aspiration alone, whereas the combination of aspiration, injection of sympathomimetic, and irrigation have a reported efficacy of 43-81% (11, 12).

While these approaches are fairly effective in the acute setting, they are typically not effective for prolonged ischemic priapism (13). Given their less invasive nature, it is generally recommended to attempt aspiration/irrigation initially. Should this fail, however, surgical intervention should be considered promptly.

## MANAGEMENT OF PROLONGED ISCHEMIC PRIAPISM

Prolonged ischemic priapism, recurrent ischemic priapism, or priapism refractory to medical management pose a more challenging task for management. For these patients, failure of non-surgical management should prompt consideration of operative intervention in order to relieving the compression syndrome (5). Surgical approaches vary widely and include distal shunts, proximal shunts, tunneling procedures, or immediate penile prosthesis implant. Each carries its own risks, benefits, and varying likelihood of resolving prolonged ischemic priapism.

### Distal Shunts

When non-surgical intervention fails, AUA guidelines recommend the use of distal shunts with or without tunneling procedures (5). The principle of these procedures is to create a connection from the corpora cavernosum to drain the stagnant deoxygenated blood and introduce fresh, oxygenated blood. Various distal corpora-glanular shunts have been described and may be categorized as either percutaneous or open shunts. Percutaneous shunts include the Winter shunt, Ebbehoj shunt, and T-shunt, while open distal shunts consist of the Al-Ghorab shunt (14-16). No studies have compared these shunts head-to-head.

A Winter's shunt involves the use of a large bore biopsy needle to create a connection between the corpora cavernosum and the glans penis (16). This can be performed multiple times in each corpora or bilaterally to maximize drainage of ischemic blood. While relatively simple and quick, there may be limited drainage due to small shunt size. The Ebbehoj shunt uses an 11-blade scalpel rather than a needle to generate larger connections from the glans to the corpora cavernosum (15). Given the larger degree of shunting, this method tends to be more effective than the Winter's shunt at achieving detumescence (8). A T-shunt involves using a 10 blade to make a ‘T’-shaped incision on the glans (17). Typically, there is quick return of abundant ischemic blood and detumescence once this shunt is created. Though quite effective, this method has associated risks of urethral injury and glans necrosis (18).

If the above attempts at percutaneous distal shunting are insufficient, open distal shunts can be considered. The Al-Ghorab shunt involves excising 1 cm of tunica albuginea from the distal portion of the corpora cavernosa (14). This tends to be the most effective distal corpora-glanular shunt, but it is also more invasive, has a higher rate of erectile dysfunction, risk of urethral injury, glans necrosis, glans numbness, and damage to dorsal arteries or dorsal nerves (8, 18).

From a physiologic standpoint, their arterial inflow increases into the corpora cavernosa after a corpora-glanular shunt is created. This "post-ischemic hyperemia" presents similarly with decreased rigidity and pain but persistence of woody fibrosis of penile on exam (5, 18). The increased cavernosal pressure can cause a small shunt—such as Winter's or Ebbehoj—to close, causing recurrent priapism (18). Signs of recurrent or increased penile rigidity and pain should raise concern, and cavernosal blood gas should be repeated; if confirmed, more invasive management is required (5). Larger T-shunts face a lower risk of post-ischemic hyperemia from shunt closure. However, collagen exposure and tunica albuginea injury-mediated activation of the clotting cascade can cause shunt thrombosis (18). The use of antiplatelet agents in the perioperative period may decrease this risk.

Success rates for distal shunts range from 65-75% in retrospective studies, while rates of *de novo* erectile dysfunction range from 14-25% (12, 19, 20). Given the real risk of developing erectile dysfunction, it bears noting that these distal shunts necessarily violate the glans penis and the distal cap of the corporal bodies. Since refractory erectile dysfunction may require penile prosthesis placement, this glans violation has the theoretic potential of increasing the risk of cylinder extrusion through the glans.

### Proximal Shunts

Proximal shunts, such as the Quackel's and Grayhack shunts, were previously considered for cases of persistent acute ischemic priapism after a failed distal shunting procedure. However, there is limited high-quality data supporting their efficacy, with only 62 documented cases from 1960 to 2020. Of these, there was significant heterogeneity with respect to patient characteristics, duration of priapism, prior aspiration/irrigation attempts, and prior attempts at distal shunting. While aggregate data from these patients revealed a successful priapism resolution rate of 77%, reported complications included urethral injury, urethrocutaneous fistulas, cavernositis, and even pulmonary embolism.18 Given the limited supporting data after over six decades, the most recent American Urological Society/Sexual Medicine Society of North America guidelines committee deems proximal shunts a historical procedure (5).

### Tunneling Procedures – The New Frontier

In cases of prolonged ischemic priapism, distal shunting may be ineffective due to the sustained hypoxia that has caused significant edema and tissue necrosis, especially at the distal portion of the corpora cavernosa (8). More recently, tunneling techniques have been described in an effort to improve outcomes in refractory cases and avoid the need for upfront penile prosthesis placement (21). This was first popularized by Dr. Arthur "Bud" Burnett who modified the Al-Ghorab shunt; once the distal corporal window has been achieved through a 2cm glans incision, a 7/8 Hegar dilator is passed several inches proximally within the corpora (10). This so-called "snake" maneuver allowed improved disruption of the ischemic coagulum, which could then be evacuated via manual compression. In the initial description, 8 of 10 patients treated for ischemic priapism using this technique achieved successful resolution of priapism with no recurrence (22). The group recently published their long-term experience with this technique and found that 92% of patients treated with distal shunt with tunneling achieved priapism resolution (24/26) despite mean priapism duration of 58.7 hours versus only 54% of those undergoing distal shunt alone (30/56, p<.001) (23). Of those with documented sexual function at follow-up, 43% (6/14) reported erections firm enough for penetration with or without the use of phosphodiesterase 5 inhibitors. Tom Lue similarly described tunneling using urethral sounds passed retrograde via a T-shunt for refractory cases where the shunt alone does not provide adequate decompression (24).

As an alternative, penoscrotal decompression was described by Morey in 2018 (25). He noted that during penile prosthesis placement for refractory ischemic priapism, detumescence was achieved during the process of corporal dilation before the device was even placed. While achieving similar disruption of the ischemic coagulum, this method avoids violation of the distal glans, which has both cosmetic and functional implications. Penoscrotal decompression is performed via a transverse penoscrotal incision similar to that used for penoscrotal penile prosthesis placement. Dissection is carried out to the corporal tunica albuginea which are then incised down to the layer of the spongy erectile tissue. Disruption of the ischemic blood is achieved by passing a metal pediatric Yankauer (or alternative dilator such as Brooks or Hegar if the former is not available) both distally and proximally in the corpora. Decompression is then achieved via manual compression of the penile shaft. In the initial description, this process was carried out unilaterally, with the expectation that deoxygenated blood could be evacuated via the septal communication between the corpora. However, there was a higher rate of early priapism recurrence (2/10) in those undergoing unilateral decompression versus 0/15 in those in whom the procedure was performed bilaterally (26). Early reports also suggest that many patients regain some degree of erectile function even after prolonged priapism (26). For those that do develop refractory erectile dysfunction, a penile prosthesis may be placed in a delayed fashion with less concern for distal erosion.

Reflecting their enhanced efficacy at resolving cases of refractory ischemic priapism, tunneling procedures are now included in the newest American Urological Society/Sexual Medicine Society of North America guidelines. Currently, the guidelines state that clinicians should consider the addition of corporal tunneling in those patients with persistent priapism after distal shunt. However, given more recent data supporting the efficacy and potential benefits of penoscrotal decompression, it is likely to be addressed in future iterations of the guidelines as suggested by thought leaders in the field (27).

No studies have been conducted comparing the efficacy of tunneling performed via a distal glans-violating approach versus a penoscrotal glans-sparing approach. Among surgeons who had performed both procedures, a recent survey suggests that penoscrotal decompression was perceived as more effective than distal tunneling procedures (6). However, this was a survey of surgeon impression and thus subject to significant potential recall bias. The likelihood of sexual function recovery was seen as similar between the two approaches. While multi-institutional comparative studies are required, these are unlikely to be performed given the relative rarity of ischemic priapism (28). For this reason, the decision on which tunneling procedure to perform should be made at the discretion of the surgeon in the absence of more definitive data (22, 24, 26).

### Early vs Delayed Penile Prosthesis Implantation

Early penile prosthesis has been widely accepted in the management of refractory or recurrent ischemic priapism (13, 29). In such cases, the vascular smooth muscle is affected by hypoxia and acidosis as soon as 6 hours following the onset of priapism (30). Left unchecked, replacement of the vascular smooth muscle with dense corporal fibrosis is inevitable. This fibrosis leads to tissue contraction and penile shortening, and also complicates placement of penile prosthesis cylinders in the delayed setting (31).

Placement of a penile prosthesis in the acute setting thus offers the opportunity to restore sexual function in a less hostile surgical environment before the corporal fibrosis develops. This also facilitates placement of full-sized cylinders, preserving penile length and bolstering patient satisfaction (32). By contrast, with delayed placement, intra-operative complications such as cross-over events and urethral injury are more frequent, and narrow cylinders may be required (33-35). Nearly 90% of subspecialty urologists perceive prosthesis placement to be more difficult after priapism, especially when performed in the delayed setting (6). The risk of device infection may also be as high as 10% for delayed placement versus 6% for acute placement (36).

Early prosthesis placement may be performed using malleable or inflatable devices, with neither clearly showing superiority (33). Malleable devices are technically easier to place, afford a lower cost to the patient, and obviate the concern for mechanical breakdown. However, the risk of distal perforation may be higher when the glans has been violated by distal shunts (36, 37). Should a malleable be placed initially, this may be revised to an inflatable device in the future (33). Others have reported excellent outcomes when inflatable devices are placed primarily (31). Inflatable options have improved concealability when deflated and higher patient satisfaction (31). Current opinions suggest that 75% of subspecialty urologists prefer using a malleable device in the acute setting, while 84% prefer inflatable devices in the delayed setting (6). Decision of which implant to use should be made jointly between surgeon and patient after thorough discussion of risks and benefits.

The data for tunneling procedures—both penoscrotal decompression and the "snake" maneuver—suggests that some patients do recover some degree of sexual function (23, 26). In these patients, placement of a penile prosthesis may represent overtreatment. In this vein, 70% of subspecialty urologists now prefer tunneling procedures for cases of prolonged ischemic priapism (duration >24 hours) while only 10% would choose up front penile prosthesis placement (6). One ongoing research question is whether MRI may determine which patients should receive upfront IPP placement versus tunneling procedures. Smooth muscle necrosis seen on MRI correlates with the development of erectile dysfunction and may be a useful tool to decide if a patient should undergo immediate IPP placement to avoid this sequela (38).

For those patients who do require delayed penile prosthesis placement, multiple techniques have been described to mitigate the associated risks. The use of vacuum erection devices pre-operatively improves blood flow to the corpora and suppresses pro-fibrotic factors, which may reduce the technical challenge of corporal dilation (39). Cavernotomes may facilitate corporal dilation, while extended corporotomies and distal counter-incisions facilitate formal corporal excavation. Both the AMS 700™ (Boston Scientific, Marlborough, MA) and the Titan (Coloplast Corporation, Minneapolis, MN) now come in narrow cylinder options which have greatly improved the ability to successfully place cylinders in hostile corpora. As with malleable device placement, these patients may be offered revision to full-sized cylinders if desired (40).

## CONCLUDING THOUGHTS

Prolonged cases of ischemic priapism are associated with increasing difficulty in achieving detumescence and significantly increased risk of erectile dysfunction. Non-surgical management may be effective in the acute setting, but in cases of refractory or prolonged priapism, distal shunts may be required. Newer tunneling procedures appear to more effectively evacuate deoxygenated blood and potentially facilitate recovery of erectile function.

While upfront penile prosthesis placement was previously considered the procedure-of-choice for cases of severe refractory priapism, there has more recently been a paradigm shift towards the utilization of tunneling procedures in the acute setting. The high efficacy of these maneuvers and the potential for sexual function recovery, potentially allow penile prostheses to be avoided in some patients; further studies are needed to investigate this hypothesis. For those ultimately requiring delayed penile prosthesis placement, the increased complication rates should be acknowledged, although multiple techniques exist to facilitate device placement. Ultimately, there is no proven algorithmic approach to the management of this challenging condition; interventional approach remains a nuanced one that depends on both patient and surgeon factors.

## COMPLIANCE WITH ETHICAL STANDARDS

This study was exempt from IRB review given its nature as a narrative review
